# Differentiation and correlation of regional uptake heterogeneity with cardiac dysfunction in biopsy-proven transthyretin amyloid cardiomyopathy using quantitative single-photon emission computed tomography/computed tomography: a single-center, cross-sectional study

**DOI:** 10.1186/s13550-025-01292-w

**Published:** 2025-08-01

**Authors:** Masakazu Tsujimoto, Hideki Kawai, Shingo Tanahashi, Masayoshi Sarai, Yasuki Asada, Hideo Izawa

**Affiliations:** 1 Department of Medical Equipment Engineering, Clinical Collaboration Unit, School of Medical Sciences, Fujita Health University, 1-98 Dengakugakubo, Kutsukake, Toyoake, Aichi 470-1192 Japan; 2 Department of Cardiology, School of Medicine, Fujita Health University, Toyoake, Japan; 3 Department of Radiology, Fujita Health University Hospital, Toyoake, Japan

**Keywords:** Amyloidosis, Single-photon emission computed tomography, Non-invasive diagnosis

## Abstract

**Background:**

Cardiac amyloidosis requires quantitative assessment using technetium-99m pyrophosphate (^99m^Tc-PYP) single-photon emission computed tomography (SPECT)/computed tomography (CT) for adequate discrimination and evaluation of disease extent. This study aimed to evaluate the utility of standardized uptake value (SUV) analysis using ^99m^Tc-PYP SPECT/CT in pathologically-confirmed transthyretin amyloid cardiomyopathy (ATTR-CM). The study also explored the relationship between local uptake heterogeneity and indicators of cardiac impairment.

**Methods:**

Forty patients diagnosed via heart biopsy and genetic analysis (20 ATTR-CM; 4 light-chain amyloidosis, 16 non-amyloidosis) were enrolled. The mean SUVs of the heart and aorta were measured using SPECT images. Discrimination performance was evaluated by comparing each SUV, the heart-to-aorta ratio (rSUV_H/Ao_), and the heart-to-contralateral-lung ratio with pathological findings serving as the gold standard. Polar maps were analyzed to assess local SUV distribution in patients with ATTR-CM. The coefficient of variation (COV) of myocardial uptake, difference score between the septum and lateral wall (%DS), base-to-apex variability, and total cardiac SUV were calculated and compared with echocardiographic parameters.

**Results:**

All metrics were significantly different between the ATTR-CM and non-amyloidosis groups. The rSUV_H/Ao_ effectively differentiated patients with ATTR-CM from those with light-chain or non-amyloidosis. Local myocardial SUV distribution correlated with impaired cardiac function. Notably, COV showed significant correlations with e' (R = 0.782) and E/e' (R =  − 0.625), linking heterogeneity to myocardial stiffness and diastolic dysfunction. Larger %DS, which predominantly reflected the ATTR-CM pattern of high septal uptake, correlated significantly with thinner walls (average wall thickness, R =  − 0.655; relative wall thickness, R =  − 0.486). As the total cardiac SUV increased, the %DS decreased (reflecting more homogeneous distribution), and global longitudinal strain worsened (R = 0.614). These observations indicated that greater impairment was associated with a higher disease burden.

**Conclusions:**

This study demonstrated that quantitative SPECT analysis provides a valuable tool for the diagnostic evaluation and differentiation of ATTR-CM. The rSUV_H/Ao_ offers high discriminatory performance. Local heterogeneity and total myocardial uptake are closely related to the disease burden and extent, as reflected by structural and functional abnormalities on echocardiography. These findings suggest potential relevance to the non-invasive assessment of these aspects of the disease at a single time point.

**Graphical abstract:**

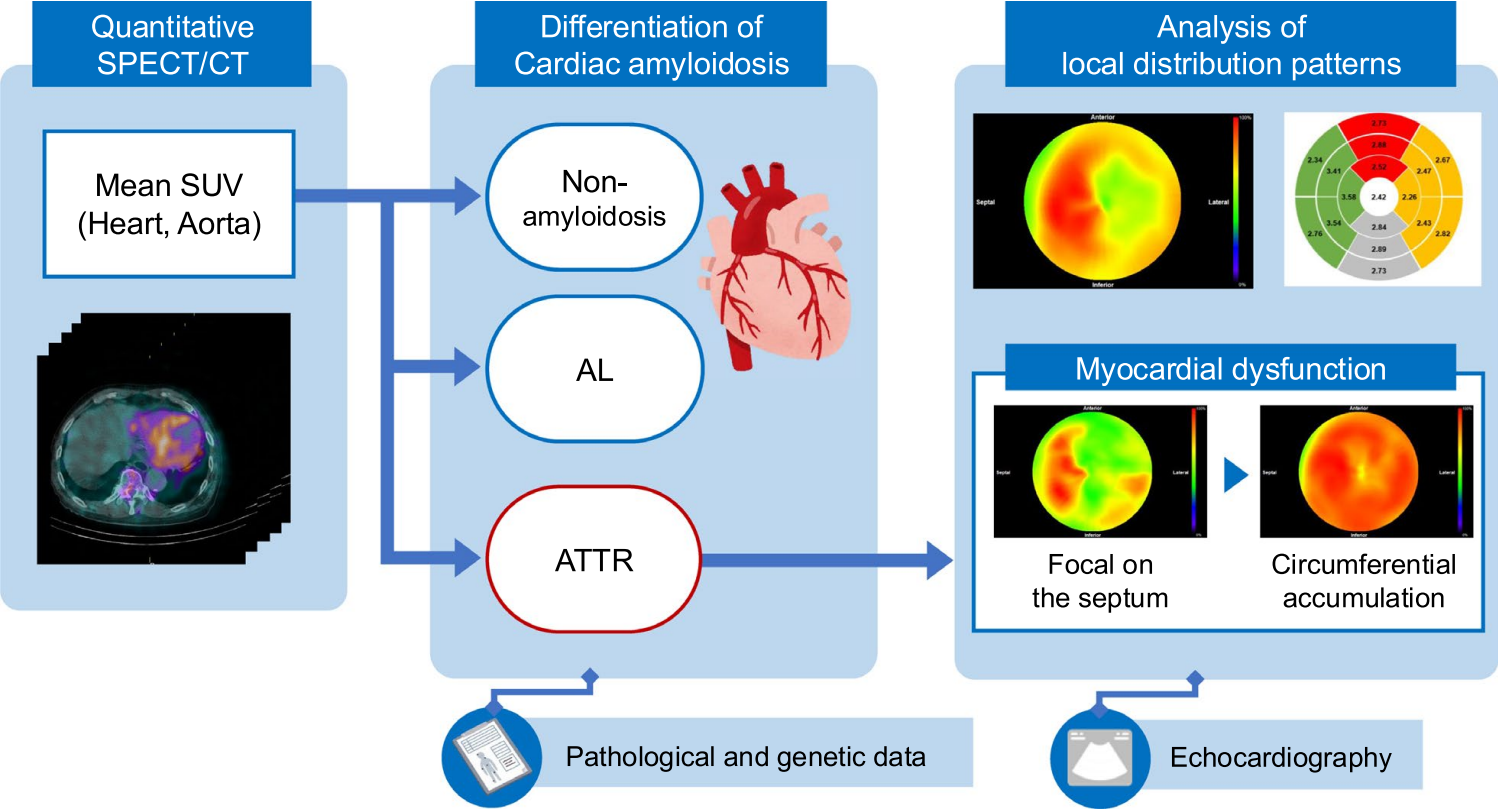

## Background

Technetium-99m-pyrophosphate (^99m^Tc-PYP) scintigraphy has become a widely used noninvasive diagnostic tool for cardiac amyloidosis, with high sensitivity and specificity for detecting transthyretin-related cardiac amyloidosis (ATTR-CM) [1–4]. A characteristic pattern of diffuse myocardial uptake observed on ^99m^Tc-PYP imaging is highly suggestive of ATTR-CM [5].

Planar (static) anterior chest images obtained using ^99m^Tc-PYP have traditionally been used for diagnostic purposes. The Perugini score [6] and heart-to-contralateral lung (H/CL) ratio are commonly used methods for assessing cardiac uptake. However, these planar imaging techniques are limited in their diagnostic accuracy due to the overlap of structures such as ribs and blood pools [7, 8]. Single-photon emission computed tomography (SPECT)/computed tomography (CT) improves differentiation between myocardial ^99m^Tc-PYP uptake and blood pool activity, thereby enabling a more precise diagnosis [7–11].

Recent software advancements have made it feasible to quantify SPECT images using the standardized uptake value (SUV), a metric commonly employed in positron emission tomography (PET) [12, 13]. When using SPECT to evaluate ATTR-CM, the regions of interest are typically defined as the myocardium and either the ribs or aorta. Previous studies have utilized SUV-derived metrics to compare ATTR-CM with H/CL ratios or other modalities [14, 15], examine associations with risk events such as heart failure or death [16], and correlate these with cardiac function tests such as echocardiography and electrocardiogram [17]. However, while many studies have investigated these quantitative values in relation to classifications based on clinical symptoms, cardiac magnetic resonance imaging (MRI) results, or genetic testing, there is limited research that has focused exclusively on pathological and genetic data. Numerous studies have explored the relationship between features derived from volumetric indices [15, 17, 18], such as cardiac pyrophosphate activity and cardiac pyrophosphate volume, which reflect the intensity and extent of uptake, respectively. However, these studies have tended to provide a global assessment of the myocardium and generally lack a quantitative interpretation of the distribution of lesions in specific regions.

The objective of this study was to investigate whether quantitative SUV measurements of myocardial and background uptake could effectively differentiate among ATTR-CM, light-chain amyloidosis (AL), and non-amyloidosis. Pathological and genetic classifications were the basis for this analysis. We also aimed to determine the correlation between the local distribution patterns, which reflected disease extent, and cardiac functional impairment.

## Methods

### Study participants

Between August 2019 and August 2024, 111 consecutive patients underwent PYP scintigraphy for suspected heart failure, cardiomyopathy, or cardiac amyloidosis. From this initial cohort, patients were included in the present study if they had a definitive diagnosis established by endomyocardial biopsy with immunohistochemical staining, supplemented by genetic testing where appropriate. This selection process yielded a final study population of 40 participants who were subsequently classified into four groups based on their diagnosis: cardiac transthyretin amyloidosis wild-type (ATTRwt), cardiac transthyretin amyloidosis variant (ATTRv), AL amyloidosis, and non-amyloidosis. Estimated glomerular filtration rate (eGFR) data were utilized, along with echocardiographic parameters obtained within one month of the ^99m^Tc-PYP scintigraphy.

### Overview of the study methods

**Fig. 1:**
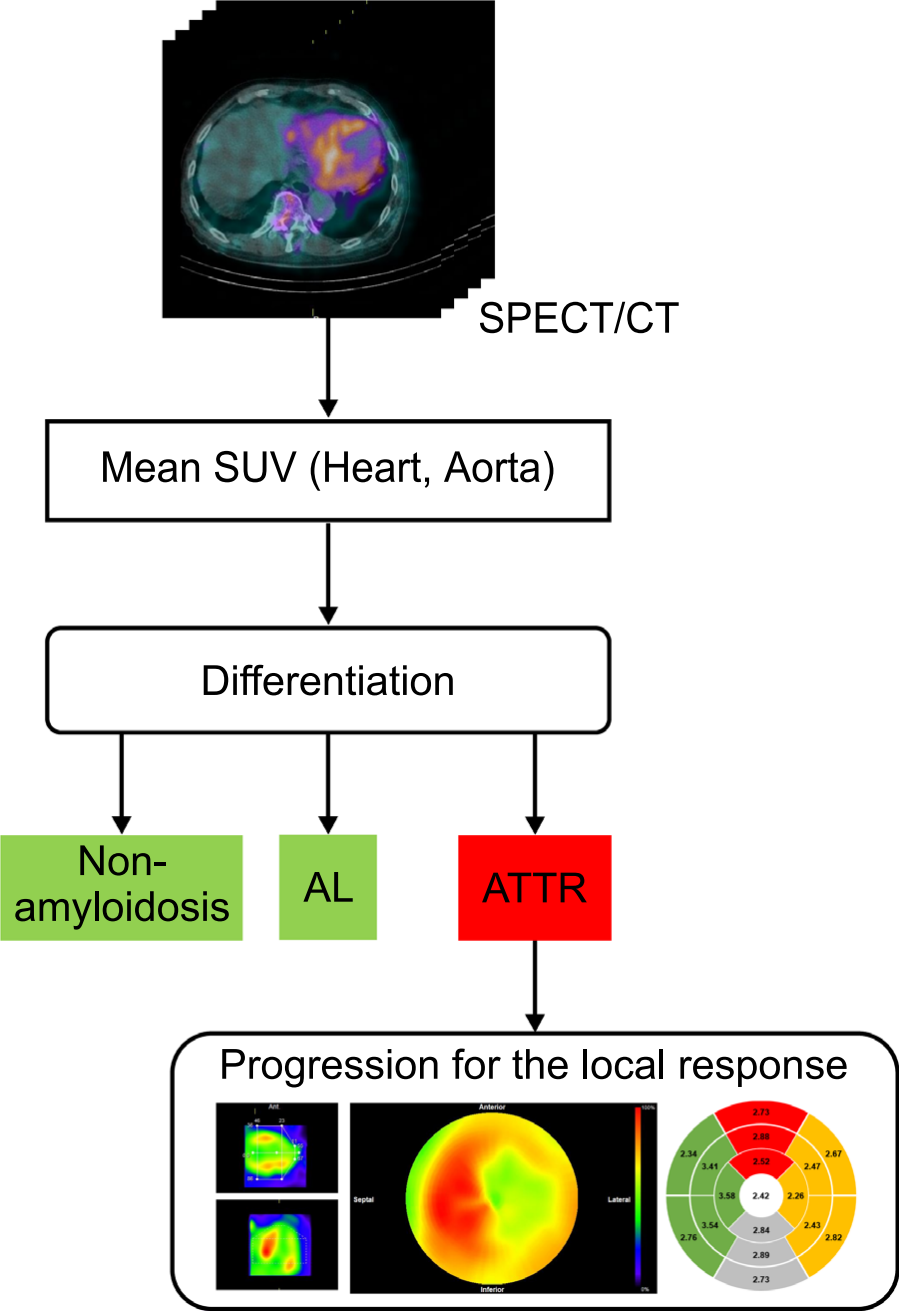
Overview of differentiation and cardiac function assessment

Figure 1 provides an overview of the methods used in this study. First, to differentiate between positive and negative ATTR-CM results, the mean SUVs of the aorta and heart were measured using SPECT/CT images. Next, following a positive differential diagnosis of ATTR-CM (either ATTRwt or ATTRv), a regional analysis was conducted to assess characteristics related to the disease extent and burden. For this regional analysis, the SPECT images of the cardiac region were converted to a polar map. Subsequently, the mean SUV and coefficient of variation (COV) were calculated for each of the 17 myocardial segments to evaluate circumferential uptake and heterogeneity. Furthermore, to assess the asymmetrical distribution, a difference score in SUV between the septal and lateral walls (%DS), and base-to-apex variability (%BA) were calculated. The relationships between these derived indices and indicators of cardiac functional impairment or morphological findings were then investigated.

### Planar and SPECT/CT imaging

^99m^Tc-PYP was administered intravenously at a radiopharmaceutical dose of 555 MBq standardized to a 60-kg body weight (i.e., 555 × body weight [kg]/60 MBq), with a maximum of 555 MBq. Planar imaging was performed at 3 h post-injection, and this was followed by SPECT/CT acquisition using Symbia T16 and T6 (Siemens K.K., Tokyo, Japan) equipped with low-energy, high-resolution collimators. The SPECT/CT acquisition and reconstruction parameters were adopted from our previous study [12], in which optimal conditions were determined by evaluating the reproducibility of quantitative SUV values and noise characteristics using phantom experiments.

Planar acquisition was performed with a 15% energy window centered at 140 keV (± 7.5%), a zoom of 1.23, a scan time of 3 min, and a matrix size of 256 × 256. The SPECT acquisition was performed with a zoom of 1.23, a scan duration of 50 s/view, an angular step of 5° over 360° (72 projections total), and a matrix size of 128 × 128. The main energy window was centered at 140 keV with a 15% width (± 7.5%). The sub-energy window for scatter correction was 7% width positioned below the main energy peak of 140 keV.

Image reconstruction was performed using Syngo MI VB 10B (Siemens K.K., Tokyo, Japan). Three-dimensional ordered subset expectation maximization was applied with 6 subsets and 25 iterations. Scatter correction was performed using the dual energy window method, and attenuation correction was performed using CT images. A Gaussian filter with a full width at half maximum of 8 mm was used for noise reduction.

The SUV, standardized by body weight, was calculated using the following formula:

$$SUV = \frac{{SPECT\;pixel\;value\left[ {\frac{{count}}{{voxel}}} \right] \times CCF\left[ {{\raise0.7ex\hbox{${\frac{{Bq}}{{mL}}}$} \!\mathord{\left/ {\vphantom {{\frac{{Bq}}{{mL}}} {\frac{{count}}{{voxel}}}}}\right.\kern-\nulldelimiterspace} \!\lower0.7ex\hbox{${\frac{{count}}{{voxel}}}$}}} \right]}}{{injected\;dose\left[ {Bq} \right]/weight\left[ g \right]}}$$

The cross-calibration factor (CCF) was pre-calculated from SPECT images acquired using a cylindrical phantom (inner diameter, 16 cm; inner height, 15 cm; volume, 3016 mL) filled with a known amount of radioactivity.

The SPECT/CT analysis software programs used were RAVAT ver1.2 (Nihon Medi-Physics Co., Ltd., Tokyo, Japan), medi + FALCON ver.1.4 (Nihon Medi-Physics Co., Ltd., Tokyo, Japan), and ImageJ (National Institutes of Health, Maryland, USA).

### Differentiation analysis

Volumes of interest (VOIs) were defined on SPECT images of the heart and aorta [19, 20]; their anatomical locations were guided by referencing co-registered CT images. The ellipsoidal VOI was set individually to include the entire heart. Cylindrical VOIs were placed at three locations along the descending aorta: the proximal portion of the descending thoracic aorta, the superior border of the liver, and proximal to the bifurcation of the common iliac artery. The height of each VOI was fixed at 19.5 mm (equivalent to five pixels). As visualized on the CT component of the fusion images, the diameter was adjusted for each subject to match the width of the aorta. The mean SUVs were calculated for each VOI. The mean SUV of the heart was defined as SUV_H_, and the average SUV from the three aortic VOIs was defined as SUV_Ao_. The ratio of the mean SUV of the heart to that of the aorta (rSUV_H/Ao_) was calculated.

### Assessment of myocardial amyloid burden and disease extent

Specific quantitative SPECT/CT metrics were used to characterize myocardial ^99m^Tc-PYP uptake in patients with ATTR-CM to reflect different aspects of the disease. Disease burden, which represented the overall magnitude of tracer accumulation, was primarily assessed using total cardiac SUV. Disease extent and heterogeneity, indicating the spatial distribution and variability of tracer uptake, were assessed using the COV, %DS, and %BA.

For regional SUV calculation, polar map images were generated using the HRV-S module of medi + FALCON. This process involved extracting the maximum count along radial lines from the center of each left ventricular short-axis slice and arranging these values concentrically. Corresponding data exported from HRV-S were then divided into 17-segments according to the American Heart Association model, and the mean and standard deviation of the SUV for each segment were calculated. Additionally, two segmentation schemes were defined for regional analysis. The first divided the heart into five regions: anterior (segments 1, 7, 13), septal (segments 2, 3, 8, 9, 14), inferior (segments 4, 10, 15), lateral (segments 5, 6, 11, 12, 16), and apical (segment 17) regions. The second scheme divided the heart into apical (segments 13–17) and basal (segments 1–12) regions.

The summed SUV for each of these predefined regions was calculated using the following formula:

$${\text{Summed SUV}}_{\textrm{region}} =\sum_{\mathrm{i}}^{\textrm{n}}{\mathrm{SUV}}_{\textrm{segment},\mathrm{i}}$$

where “region” denoted one of the predefined segmented myocardial areas (e.g., septal, basal), “i” was the segment index within that area, and “n” was the total number of segments in that specific area.

Total cardiac SUV, an index of overall disease burden, was calculated by summing the SUVs of all 17 myocardial segments.

Several indices were calculated to assess regional uptake heterogeneity and disease extent.

First, a segmental COV was calculated for each of the 17 myocardial segments:

$$\text{Segmental COV}=\frac{\text{standard deviation of SUV within segment}}{\text{mean SUV of segment}} \times 100$$

The sum of the 17 segmental COVs was used as the COV throughout this study.

The %DS was calculated as:

$$\mathrm{DS} =\frac{\mathrm{Summed\ SUV}_{\mathrm{septal}} - \mathrm{Summed\ SUV}_{\mathrm{lateral}}}{\mathrm{Summed\ SUV}_{\mathrm{All}}} \times 100$$

The %BA was calculated as:

$$\mathrm{BA} =\frac{\mathrm{Summed\ SUV}_{\mathrm{basal}} - \mathrm{Summed\ SUV}_{\mathrm{apical}}}{\mathrm{Summed\ SUV}_{\mathrm{All}}} \times 100$$

### Evaluation metrics

Using histopathological findings and genetic test results, differentiation analysis was performed to categorize the patients into three groups: ATTR-type (ATTRwt and ATTRv), AL-type, and negative control group (non-amyloidosis). The SUV_Ao_, SUV_H_, rSUV_H/Ao_, and H/CL ratios were compared among the groups.

For patients in the ATTR-CM group (ATTRwt and ATTRv), four SPECT-derived indices were calculated to assess their potential to reflect disease extent and burden: COV, %DS, %BA, and total cardiac SUV. These indices were then correlated with the following echocardiographic parameters to assess myocardial impairment. Morphological parameters included average wall thickness (AWT), defined as the average of interventricular septal thickness and left ventricular posterior wall end-diastolic thickness; relative wall thickness (RWT), calculated by dividing the posterior wall thickness by the left ventricular end-diastolic diameter. Functional parameters included e' as a marker of myocardial relaxation related to myocardial stiffness; E/e' as an index of diastolic function; and global longitudinal strain (GLS) as a measure of global systolic function. Echocardiography was performed using a Vivid E95 (GE Healthcare., K.K., Tokyo, Japan). Cases with heart rate variability exceeding 10% were excluded from the GLS analysis.

### Statistical analyses

Statistical analyses were performed using Bell Curve for Excel (Social Information Service Inc., Tokyo, Japan). Continuous variables were presented as mean ± standard deviation or median [interquartile range]. Categorical variables were presented as percentages.

Comparisons of baseline characteristics (age, eGFR) across the four study groups (ATTRwt, ATTRv, AL, and non-amyloidosis) and quantitative imaging metrics (mean SUV, rSUV_H/Ao_, and H/CL ratios) across three primary diagnostic groups (ATTR [ATTRwt and ATTRv combined], AL, and non-amyloidosis) were performed using the Kruskal–Wallis test, which was chosen due to the potential for small sample sizes and non-normal distribution within some groups. If a significant overall difference was detected by the Kruskal–Wallis test, post-hoc pairwise comparisons were conducted using the Steel–Dwass test. Sex distribution across the four primary groups was assessed using Fisher’s exact test. If a significant overall difference was indicated, pairwise comparisons between groups were performed with Bonferroni correction for multiple comparisons.

Pearson’s correlation analysis was conducted to investigate the correlation between COV, %DS, %BA, total cardiac SUV, and echocardiographic parameters (AWT, RWT, e', E/e', and GLS). The significance level was set at *p* < 0.05 (two-tailed). This study was approved by the Fujita Health University Institutional Review Board (approval number: HM24-490), and all image data were anonymized.

## Results

### Patient characteristics

A total of 40 patients were included in this study: 15 with ATTRwt amyloidosis, 5 with ATTRv, 4 with AL amyloidosis, and 16 with non-amyloidosis. The baseline characteristics of the study participants are summarized in Table 1.

**Table 1:** Patient characteristics

Characteristic	Nonamyloidosis (*n* = 16)	ATTRwt (*n* = 15)	ATTRv (*n* = 5)	AL (*n* = 4)	Total (*n* = 40)
Age [years] (mean ± SD)	61 ± 18	77 ± 5*	75 ± 3	71 ± 12	70 ± 14
Male n (%)	11 (68. 8%)	15 (100%)	3 (60%)	4 (100%)	33 (82.5%)
eGFR [mL/min/1.73m^2^] (median ± IQR)	43.3 ± 21.6	36.6 ± 27.6	63.1 ± 11.4^†^	64.9 ± 10.6	45.3 ± 30.7

AL, light-chain amyloidosis; ATTRwt, cardiac transthyretin amyloidosis wild-type; ATTRv, cardiac transthyretin amyloidosis variant; eGFR, estimated glomerular filtration rate; IQR, interquartile range; SD, standard deviation

*P-value < 0.05 vs. Non-amyloidosis group. ^†^ *P*-value < 0.05 vs. Non-amyloidosis group

The mean age of the entire cohort was 70 ± 14 years. Patients in the ATTRwt group (77 ± 5 years) were significantly older than those in the non-amyloidosis group (61 ± 18 years, *p* = 0.010). A significant difference in sex distribution was found among the four groups (ATTRwt, ATTRv, AL, and non-amyloidosis) using Fisher’s exact test (*p* = 0.037). However, after applying the Bonferroni correction for multiple comparisons, no significant differences in sex distribution were observed in the pairwise comparisons between key diagnostic groups (non-amyloidosis vs. ATTRwt, *p* = 0.130; non-amyloidosis vs. AL, *p* = 1.000; ATTRwt vs. ATTRv, *p* = 0.158). Regarding renal function, eGFR was significantly higher in the ATTRv group than in the non-amyloidosis group (*p* = 0.044).

### Differentiation analysis

**Fig. 2:**
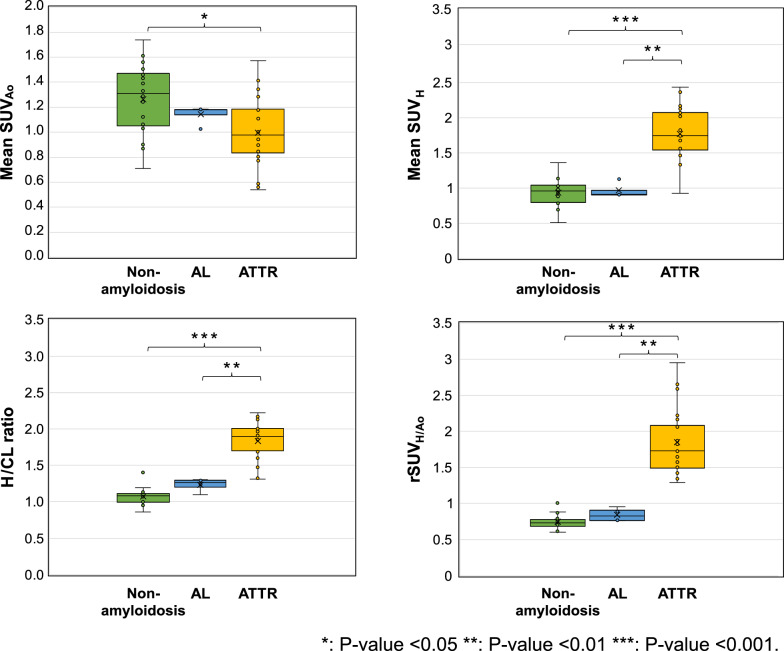
Comparison of quantitative SPECT/CT metrics and H/CL ratio across pathological groups. Box plots comparing key quantitative metrics derived from SPECT/CT images and the H/CL ratio among patients with transthyretin amyloid cardiomyopathy (ATTR-CM), amyloid light-chain (AL) amyloidosis, and non-amyloidosis controls. Metrics shown include mean cardiac SUV (SUV_H_), mean aortic SUV (SUV_Ao_), and heart-to-aorta SUV ratio (rSUV_H/Ao_) derived from SPECT/CT, and the heart-to-contralateral lung ratio (H/CL) derived from 3-h planar images. Whiskers extend to 1.5 times the interquartile range (IQR), boxes represent the IQR, and the central line indicates the median value. Mean values are indicated by 'x' markers. Statistical significance for inter-group comparisons was assessed using the Kruskal–Wallis test followed by the Steel–Dwass test for post-hoc multiple comparisons. Significance levels are indicated by asterisks as defined in the legend within the figure

Figure 2 illustrates the relationship between the mean SUV in each region, the H/CL ratio, and the rSUV_H/Ao_. Pathological findings were used as the reference standard. Significant differences were observed between the ATTR and non-amyloidosis groups in all comparisons (*p* < 0.001, except for the aorta, where *p *= 0.026). Furthermore, when comparing the ATTR and AL groups, SUV_H_, H/CL ratio, and rSUV_H/Ao_ were all significantly higher in the ATTR group (*p* < 0.01 for all three comparisons; Fig. 2). However, SUV_Ao_ was significantly lower in the ATTR group (1.00 ± 0.28) than in the non-amyloidosis group (1.27 ± 0.29), and SUV_H_, H/CL ratio, and rSUV_H/Ao_ were significantly higher in the ATTR group compared to those in both the AL and non-amyloidosis groups. The SUV_H_ was 1.76 ± 0.38 in the ATTR group, 0.96 ± 0.11 in the AL group, and 0.93 ± 0.20 in the non-amyloidosis group. Similarly, H/CL ratios were 1.84 ± 0.26, 1.23 ± 0.09, and 1.07 ± 0.12, in the ATTR, AL, and non-amyloidosis groups, respectively, and rSUV_H/Ao_ values were 1.85 ± 0.47, 0.84 ± 0.09, and 0.75 ± 0.11, respectively.

Using a cutoff of 1.3 for the H/CL ratio, one of the 16 non-amyloidosis cases yielded a false positive. In the AL group, only one patient had an H/CL ratio of 1.3. In contrast, when using an rSUV_H/Ao_ cutoff between 1.1 and 1.3, the ATTR group was successfully differentiated from the non-amyloidosis and AL groups with 100% sensitivity and specificity.

### Assessment of myocardial amyloid burden and disease extent

**Fig. 3:**
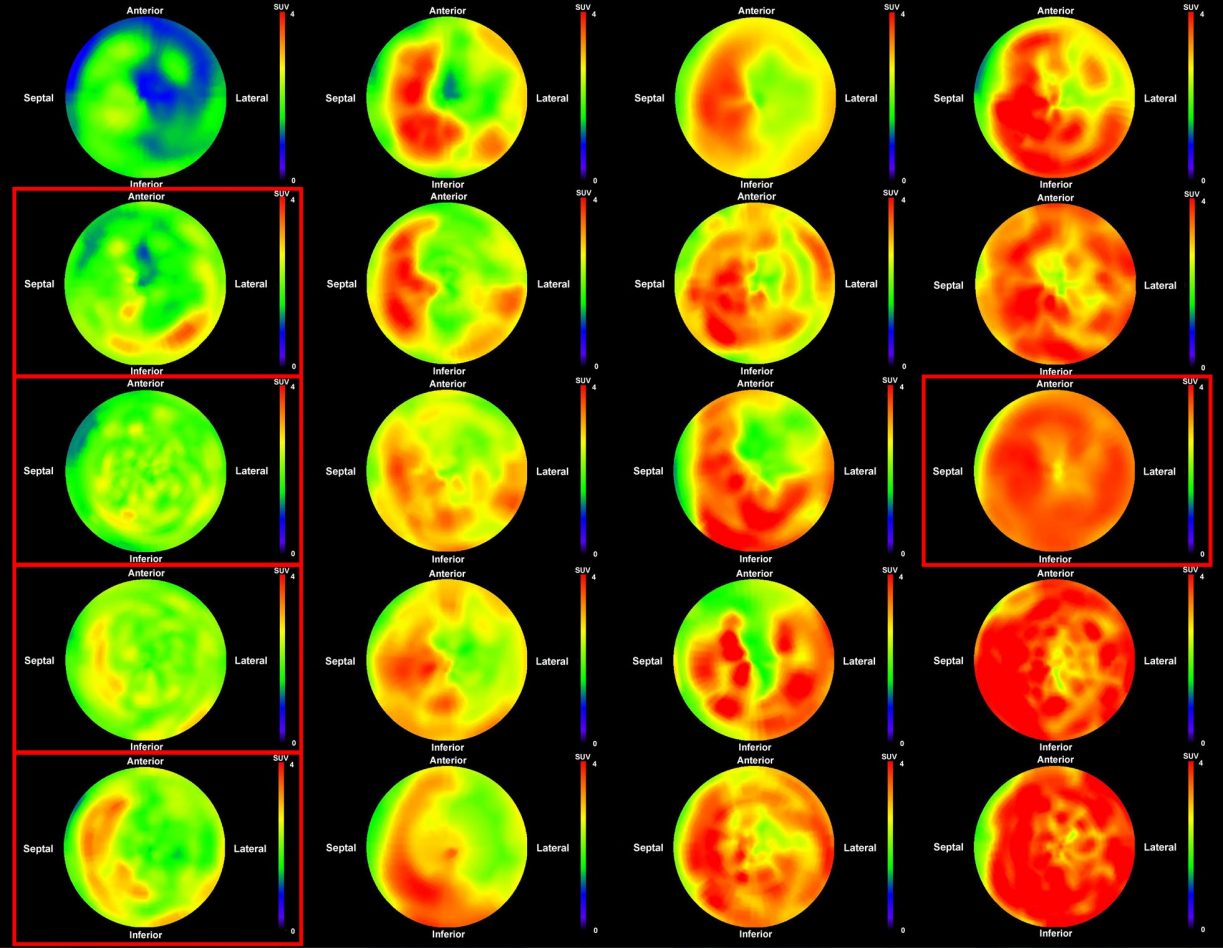
Polar maps of myocardial ^99m^Tc-PYP uptake in patients with transthyretin amyloid cardiomyopathy. Polar maps displaying regional SUV distribution in all 20 patients with transthyretin amyloid cardiomyopathy, sorted by ascending total cardiac SUV (top-left = lowest, bottom-right = highest) to visualize patterns associated with increasing amyloid burden. Red frames indicate cardiac transthyretin amyloidosis variant cases; unframed maps represent cardiac transthyretin amyloidosis wild-type. The upper SUV display limit is set to 4.0 for all maps. The center of the polar map represents the apex

Figure 3 shows the polar maps of all 20 ATTR-positive cases identified in the differentiation analysis. High-uptake regions were frequently observed in the septum and basal lateral wall, whereas uptake in the apical lateral wall was typically low. As the total cardiac SUV increased, high-uptake regions expanded in the septum and basal lateral wall, while apical lateral wall uptake gradually increased. Table 2 presents the results of the local analysis for each myocardial region.  As the total cardiac SUV increased, SUV_septal_ and SUV_lateral_ increased similarly, but SUV_basal_ increased more steeply than SUV_apical_.

**Table 2:** Measurement results from local response analysis

Type	Total cardiac SUV	COV	Summed SUV_region_			
			Basel	Apical	Septal	Lateral
wt	24.16	259.40	17.23	6.93	8.64	6.19
v	33.46	157.07	24.65	8.81	9.61	10.47
v	35.99	161.16	25.49	10.50	10.66	10.30
v	37.42	155.92	26.70	10.72	11.71	10.77
v	38.23	221.59	28.08	10.15	12.75	10.34
wt	44.36	285.17	31.73	12.63	14.80	12.04
wt	45.39	211.71	32.96	12.43	16.21	13.01
wt	46.01	153.64	32.91	13.10	14.44	13.01
wt	46.38	166.83	33.05	13.33	15.07	12.04
wt	46.49	159.86	32.86	13.64	14.56	11.45
wt	47.29	157.07	33.67	13.62	15.64	12.64
wt	50.12	314.87	36.47	13.65	15.88	16.11
wt	50.17	186.86	35.19	14.98	16.08	13.69
wt	50.30	239.24	36.32	13.98	15.73	13.71
wt	53.61	165.70	38.66	14.95	16.75	15.55
wt	55.20	204.80	39.04	16.16	17.60	14.40
wt	56.35	148.96	40.66	15.69	17.22	16.26
v	58.37	86.38	41.39	16.99	17.31	17.16
wt	62.72	172.45	45.66	17.06	20.09	17.67
wt	62.77	176.56	44.83	17.94	18.27	18.49

*COV* coefficient of validation, *SUV* standardized uptake value, *wt* wild-type, *v* variant

**Fig. 4:**
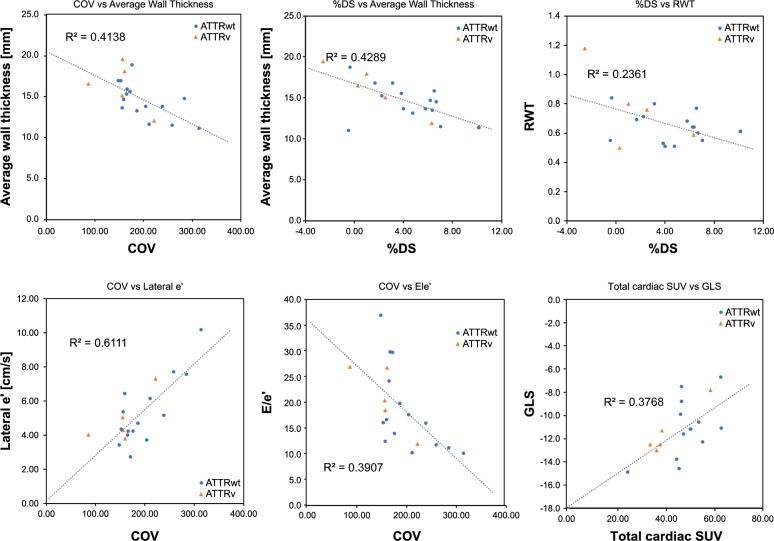
Correlation between regional SUV indices and echocardiographic parameters

Table 3 shows the correlation between the echocardiographic findings and local SUV analysis. Figure 4 illustrates the relationship between the echocardiographic parameters and SUV indices obtained from the local analysis. Among the morphological indices, AWT showed a significant negative correlation with COV (R = -0.643, *p* = 0.002) and %DS (R = -0.655, *p* = 0.002). The RWT was negatively correlated with %DS (R = -0.486, *p* = 0.030). Among the parameters reflecting myocardial stiffness and diastolic function, lateral e' was correlated only with COV (R = 0.782, *p* < 0.001), and septal e' showed a significant positive correlation with COV (R = 0.600, *p* = 0.005). In addition, COV was negatively correlated with E/e' (R = -0.625, *p* = 0.003). Among the indices of systolic function, GLS correlated with COV (R = -0.596, *p* = 0.009) and total cardiac SUV (R = 0.614, *p* = 0.007).

**Table 3:** Relationship between regional SUV parameter and electrocardiogram

Electrocardiogram		COV		%DS		%BA		Total cardiac SUV	
		R	P	R	P	R	P	R	P
AWT	n = 20	− 0.643	0.002**	− 0.655	0.002**	− 0.036	0.88	0.193	0.414
RWT	n = 20	− 0.283	0.227	− 0.486	0.030*	0.223	0.34	− 0.311	0.182
GLS	n = 18	− 0.596	0.009**	− 0.208	0.408	− 0.066	0.80	0.614	0.007**
Septal e'	n = 20	0.600	0.005**	0.110	0.644	0.376	0.10	− 0.283	0.227
Lateral e'	n = 20	0.782	0.000***	0.276	0.239	0.197	0.41	− 0.420	0.065
E/e' (Ave)	n = 20	− 0.625	0.003**	− 0.301	0.197	− 0.118	0.62	0.346	0.135

COV, coefficient of validation; %DS, difference score between the septal and lateral walls; %BA, base-to-apex variability; SUV, standard uptake value; AWT, average wall thickness; RWT, Relative wall thickness; GLS, global longitudinal strain.

*P-value <0.05, **P-value <0.01, ***P-value <0.001

**Fig. 5:**
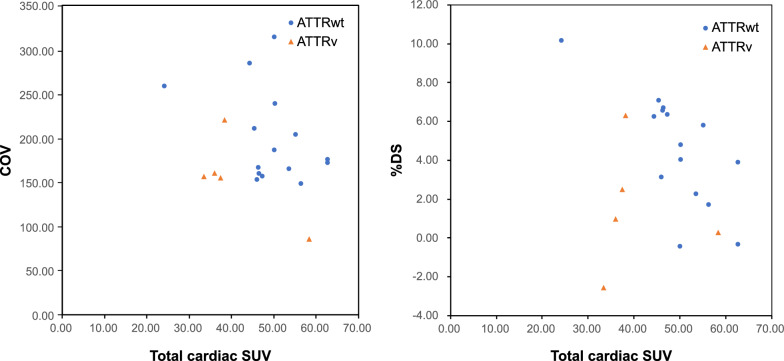
Relationship between total cardiac SUV (burden) and regional heterogeneity metrics (extent)

Table 4 and Fig. 5 present the relationships between the total cardiac SUV and local SUV variability, respectively. When both ATTRwt and ATTRv were analyzed, no significant correlations were found between total cardiac SUV and %DS, %BA, or COV. However, when the analysis was limited to ATTRwt, a significant negative correlation was observed between %DS and total cardiac SUV(R =  − 0.728, *p* = 0.002).

**Table 4:** Relationship between regional SUV parameters for total cardiac SUV

		COV		%DS		%BA	
		R	P	R	P	R	P
ATTRwt + ATTRv	n = 20	− 0.254	0.280	− 0.295	0.206	-0.100	0.676
ATTRwt only	n = 15	− 0.385	0.156	− 0.728	0.002**	0.226	0.417
ATTRv only	n = 5	− 0.740	0.153	− 0.039	0.951	-0.486	0.406

COV, coefficient of validation; %DS, difference score between the septal and lateral walls; %BA, base-to-apex variability; ATTRwt, cardiac transthyretin amyloidosis wild-type; ATTRv, cardiac transthyretin amyloidosis variant

**P-value < 0.01

## Discussion

This study utilized quantitative SUV analysis to investigate the discrimination performance of ^99m^Tc-PYP SPECT/CT for ATTR-CM in a robust cohort of patients with histopathologically and genetically confirmed cardiac amyloidosis. We also evaluated the relationship between regional analysis of myocardial ^99m^Tc-PYP uptake patterns derived from SPECT/CT and cardiac function in the context of disease burden and extent.

### Differentiation analysis

A significant difference was found between the ATTR and non-amyloidosis groups for SUV_H_, SUV_Ao_, rSUV_H/Ao_, and H/CL ratio. The elevated cardiac SUV observed in the ATTR group supports the hypothesis that PYP specifically binds to amyloid deposits in the myocardium. This is consistent with the notion that ^99m^Tc-PYP binds to calcium deposits as well as accumulates in areas of amyloid protein deposition [21]. Matsuda et al. [15] reported a significantly higher SUV_H_ of 1.9 ± 0.5 in the ATTR group compared to 1.1 ± 0.2 in the non-amyloidosis group when using a 40% fixed threshold, while Dorbala et al. [22] reported values of 2.4 ± 0.6 and 1.3 ± 0.3, in the ATTR and non-amyloidosis groups, respectively. Differences in the VOI definition might have accounted for this discrepancy, as Matsuda et al. defined the VOIs based on high-uptake areas, whereas Dorbala et al. used the myocardial wall of the left ventricle. Our simpler approach, encompassing the entire myocardium and blood pool, yielded quantitative results in non-ATTR groups comparable to these previous studies [15, 22]. Given the potential for an elevated SUV_H_ in regions with localized uptake and the unpredictable nature of amyloid deposition, we adopted a more inclusive approach by evaluating the entire heart. Our results demonstrated that the mean SUV of the entire myocardium in cases of ATTR-CM might have overlapped among the ATTR, AL, and non-amyloidosis groups, suggesting a potential trade-off between sensitivity and specificity.

Despite considering factors such as calcification and blood pool variability, the reason for the lower SUV_Ao_ in our study remains unclear. Several factors might contribute: First, the pronounced myocardial uptake of ^99m^Tc-PYP in ATTR-CM could lead to a relatively lower tracer concentration in the systemic circulation, thereby reducing SUV_Ao_. Second, we considered the influence of potential confounders. While the ATTRv subgroup showed better renal function (eGFR) than that of the non-amyloidosis group (*p* < 0.05), no significant difference was found between the non-amyloidosis group and the ATTRwt subgroup (which comprised most of the ATTR cohort) (*p* = 0.813). Thus, differences in renal function were unlikely to be the primary driver for the lower SUV_Ao_ in the ATTR group. Despite the significant age difference between the ATTRwt subgroup and the non-amyloidosis group, a direct causal link between older age and lower SUV_Ao_ was not immediately apparent, though it might reflect unmeasured factors. The dark blood pool phenomenon is a characteristic finding in patients with ATTR-CM on delayed contrast-enhanced cardiac MRI and is caused by the distribution of contrast within amyloid deposits [23, 24]. This mechanism may similarly contribute to reduced SUV_Ao_ with ^99m^Tc-PYP, as potentially observed in this study. However, the overlapping SUV values among the ATTR, AL, and non-amyloidosis groups in both the heart and aorta highlight the limitations of using a single SUV index for a definitive diagnosis.

With a cutoff value > 1, the rSUV_H/Ao_ demonstrated 100% sensitivity and specificity in differentiating between patients with ATTR amyloidosis and those in the non-amyloidosis group. Compared to traditional planar methods, our quantitative SPECT/CT-based rSUV_H/Ao_ showed advantages. The Perugini score, for instance, relies on subjective visual interpretation [6]. The planar heart-to-contralateral lung (H/CL) ratio (typically using a cutoff of ≥ 1.3 at 3 h post-injection) offers a more quantitative planar measure [7, 8], but in our cohort, it yielded a 6.3% false-positive rate in the non-amyloidosis group, likely due to blood pool contamination. Notably, the single false-positive H/CL case (an 81-year-old with low eGFR of 18.5 mL/min/1.73m^2^) still had a low rSUV_H/Ao_. This finding underscores the robustness of the rSUV_H/Ao_ metric. Although renal impairment can elevate blood pool activity [25] and is common in AL amyloidosis [7, 26, 27], the precise cause for this patient's elevated H/CL ratio remains unclear, as no overall correlation between H/CL ratio and age or eGFR was found in our non-amyloidosis cohort. Crucially, even AL amyloidosis patients who exceeded the H/CL cutoff of 1.3 exhibited low rSUV_H/Ao_. The rSUV_H/Ao_ also appeared less susceptible to blood pool contamination, potentially due to the tomographic nature of SPECT and the direct aortic blood pool reference. The results of this study suggest that rSUV_H/Ao_ may be a highly accurate, non-invasive biomarker capable of guiding clinical decisions and potentially reducing the need for invasive testing in select patient populations.

### Assessment of myocardial amyloid burden and disease extent

Left ventricular hypertrophy in amyloidosis is associated with increased myocardial stiffness, which leads to impaired basal contractility [28]. In this condition, diastolic dysfunction is often present before systolic dysfunction [7]. Our results showed that COV, an indicator of uptake heterogeneity that reflects disease extent, was correlated with myocardial wall thickness, e', and E/e'. As local variability decreased, the myocardium became thicker and stiffer and diastolic function worsened, which suggested a more severe cardiac impairment. Table 2 indicates that, while the SUV_septal_ and the SUV_lateral_ increased similarly with a higher overall myocardial SUV (representing increased disease burden), the SUV_basal_ increased more steeply than the SUV_apical_. This observation indicated changing patterns of disease extent. These findings suggest that heterogeneity in local PYP uptake, reflecting disease extent, was prominent in cases with lower disease burden, potentially representing early patterns. This heterogeneity appeared to lessen in cases with higher disease burden due to a more uniform concentric deposition, primarily in the basal region, leading to a decrease in COV.

The greatest contributing factor to the variability in COV, especially in more heterogeneous cases (potentially reflecting lower disease burden/extent), was the tendency for higher PYP uptake in the septum relative to the lateral apex, as shown in Fig. 3. Although no detailed pathological reports have compared amyloid deposition in different myocardial regions, prior studies using visual assessment [29], comparison of uptake differences [30], and count ratios [31, 32] consistently show higher septal uptake. Ogasawara et al. reported that a lower PYP uptake in the septum relative to the lateral wall was associated with adverse outcomes in patients with heart failure or cardiac death [31]. Grigoratos et al. hypothesized that amyloid deposition preferentially involves the region from the septum to the inferior wall, followed by the basal to apical region [32]. Although our results did not reveal a significant correlation with the %BA, which is a typical finding in amyloidosis [33], the increased rate of the SUV_basal_ compared to that of the SUV_apical_, coupled with the decreasing %DS (Table 2, Figs. 4, 5), suggested that the differential uptake between the septum and lateral wall indirectly reflected the pattern and extent of amyloid deposition within the myocardium at the time of imaging.

In our study, the GLS deteriorated as the total cardiac SUV increased and the COV decreased. This suggested that higher PYP uptake, which reflects greater amyloid deposition, was associated with impaired myocardial contractile function [34]. Watanabe et al. found that GLS was correlated with volumetric measures of amyloid deposition [35]. Our approach differed from that study in that we utilized total cardiac SUV for burden and polar map-derived metrics to determine altered uptake distribution (disease extent). However, our results did suggest that impaired contractility was linked to both increased myocardial amyloid burden and altered extent. Moreover, the correlation we observed between GLS and COV was likely attributable to the known associations between uptake heterogeneity, myocardial stiffness, and diastolic dysfunction.

In summary, our findings collectively support the hypothesis that the pattern of PYP uptake heterogeneity (COV, %DS) reflects the extent, and potentially the stage, of amyloid deposition. Early/less extensive disease may present with more heterogeneous uptake and pronounced %DS, particularly as observed in patients with ATTRwt in whom a lower total cardiac SUV was correlated with a higher %DS, linking to morphological and diastolic changes. Conversely, with increasing amyloid burden (higher total cardiac SUV), PYP uptake tended to become homogeneous throughout the myocardium, evidenced by decreased COV and reduced %DS in ATTRwt, implying more diffuse myocardial involvement in advanced stages of disease. However, Fig. 5 reveals some discordance between genotype, overall uptake (burden), and heterogeneity (extent) in a subset of cases, which suggests that factors such as different mutations might have contributed to variable deposition patterns.

To place these SPECT-derived heterogeneity findings into context with other imaging modalities, PET imaging is an emerging tool for ATTR-CM, primarily quantifying overall cardiac tracer uptake (e.g., SUV, target-to-background ratio) [36]. However, a recent systematic review highlighted that assessing spatial distribution or heterogeneity of amyloid deposition with PET often still relies on subjective visual interpretation, making robust quantitative analysis for monitoring a key challenge [37]. In contrast, our study demonstrated that widely accessible SPECT/CT provided quantitative metrics of regional ^99m^Tc-PYP uptake heterogeneity (e.g., COV, %DS) that correlated with cardiac dysfunction and amyloid burden. This quantitative SPECT-based approach to spatial characteristics offers a potentially valuable complement to overall uptake quantification and represents an initial step toward a more detailed characterization of cardiac amyloidosis.

### Study limitations and clinical perspectives

The limitations of this study included its single-center design and relatively small sample size, which might have introduced bias. The AL amyloidosis cohort was particularly small (n = 4). While these patients were included in initial SUV and H/CL ratio comparisons, they exhibited low overall cardiac uptake, consistent with the typical findings for AL amyloidosis on ^99m^Tc-PYP imaging. Consequently, detailed regional uptake analyses (e.g., COV, %DS, %BA) were restricted to ATTR-CM patients, thus warranting dedicated studies with larger AL cohorts to explore their regional uptake characteristics using different tracers. Furthermore, the cross-sectional design utilized data only taken from a single time point, and this precluded the evaluation of disease progression within individual patients. Despite these limitations, robust patient selection (pathologically- and genetically-confirmed cases) and the observed correlations between uptake heterogeneity and echocardiographic measures of myocardial structure and function underscore the potential clinical relevance. Future multicenter, longitudinal studies with larger cohorts are needed to validate and expand upon our findings.

This study demonstrates that quantitative SPECT analysis provides a nuanced view of ATTR-CM beyond simple diagnosis. We established that the rSUV_H/Ao_ is a robust metric for differentiation, while regional heterogeneity indices, such as COV and %DS, correlate significantly with echocardiographic measures of myocardial structure and function. These findings strongly suggest that these SPECT-derived metrics reflect not only the spatial distribution of amyloid but also its pathophysiological impact on cardiac mechanics. This multi-faceted quantitative approach opens promising avenues for future clinical application. For instance, COV and %DS could potentially serve as biomarkers for early risk stratification, identifying subtle, heterogeneous amyloid patterns before overt global dysfunction emerges. Furthermore, tracking changes in these heterogeneity metrics over time may offer an objective means of monitoring response to amyloid-modifying therapies. Such applications, pending prospective validation in larger, longitudinal studies, could aid in developing more personalized treatment strategies for patients with ATTR-CM.

## Conclusions

In patients with suspected cardiac amyloidosis who underwent ^99m^Tc-PYP SPECT/CT, quantitative assessment of myocardial uptake based on pathological and/or genetic classification offered superior diagnostic performance to two-dimensional planar analysis. Additionally, the ratio of cardiac-to-aortic uptake further improved diagnostic accuracy. Quantitative regional assessment was associated with left ventricular wall thickness and the degree of myocardial dysfunction in patients with ATTR-CM, suggesting its potential utility in assessing cardiac function.

## Data Availability

Not applicable.

## Abbreviations

AL: Amyloid light-chain
ATTR-CM: Transthyretin amyloid cardiomyopathy
ATTRv: Cardiac transthyretin amyloidosis variant
ATTRwt: Cardiac transthyretin amyloidosis wild-type
%BA: Base-to-apex variability
COV: Coefficient of variation
%DS: Difference score between the septal and lateral walls
GLS: Global longitudinal strain
H/CL: Heart to contralateral lung ratio
rSUV_H/Ao_: SUV ratio of heart to aorta
RWT: Relative wall thickness
SUV: Standardized uptake value
SUV_Ao_: Average SUV from the three aortic VOIs
SUV_H_: Mean SUV of the heart
^99^^m^Tc-PYP: Technetium-99m pyrophosphate
VOI: Volume of interest
