# Correction: Differentiation and correlation of regional uptake heterogeneity with cardiac dysfunction in biopsy-proven transthyretin amyloid cardiomyopathy using quantitative single-photon emission computed tomography/computed tomography: a single-center, cross-sectional study

**DOI:** 10.1186/s13550-026-01371-6

**Published:** 2026-03-19

**Authors:** Masakazu Tsujimoto, Hideki Kawai, Shingo Tanahashi, Masayoshi Sarai, Yasuki Asada, Hideo Izawa

**Affiliations:** 1 Department of Medical Equipment Engineering, Clinical Collaboration Unit, School of Medical Sciences, Fujita Health University, 1-98 Dengakugakubo, Kutsukake, Toyoake, 470-1192 Aichi Japan; 2 Department of Cardiology, School of Medicine, Fujita Health University, Toyoake, Japan; 3 Department of Radiology, Fujita Health University Hospital, Toyoake, Japan

**Correction: EJNMMI Research (2025) 15:97**

**https://doi.org/10.1186/s13550-025-01292-w**

Following publication of the original article, Fig. 2 has been corrected: owing to formatting issues, not all information provided by the figure was clearly legible. The authors and publisher thank you for reading this erratum.

